# Multimodal Approach to Neurocognitive Function in People Living with HIV in the cART Era: A Comprehensive Review

**DOI:** 10.3390/life14040508

**Published:** 2024-04-15

**Authors:** Charalampos D. Moschopoulos, Evangelia Stanitsa, Konstantinos Protopapas, Dimitra Kavatha, Sokratis G. Papageorgiou, Anastasia Antoniadou, Antonios Papadopoulos

**Affiliations:** 1 4th Department of Internal Medicine, Medical School of Athens, National and Kapodistrian University of Athens, Attikon University Hospital, 12462 Athens, Greece; kprotopapas@hotmail.com (K.P.); dimitra.kavatha@gmail.com (D.K.); ananto@med.uoa.gr (A.A.); antipapa@med.uoa.gr (A.P.); 2 1st Department of Neurology, Medical School of Athens, National and Kapodistrian University of Athens, Eginition Hospital, 11528 Athens, Greece; eva.st.92@gmail.com (E.S.); sokpapa@med.uoa.gr (S.G.P.)

**Keywords:** HIV, neurocognitive impairment, biomarkers, neuroimaging, HIV-associated brain injury

## Abstract

Combination antiretroviral treatment (cART) has revolutionized the management of human immunodeficiency virus (HIV) and has markedly improved the disease burden and life expectancy of people living with HIV. HIV enters the central nervous system (CNS) early in the course of infection, establishes latency, and produces a pro-inflammatory milieu that may affect cognitive functions, even in the cART era. Whereas severe forms of neurocognitive impairment (NCI) such as HIV-associated dementia have declined over the last decades, milder forms have become more prevalent, are commonly multifactorial, and are associated with comorbidity burdens, mental health, cART neurotoxicity, and ageing. Since 2007, the Frascati criteria have been used to characterize and classify HIV-associated neurocognitive disorders (HAND) into three stages, namely asymptomatic neurocognitive impairment (ANI), mild neurocognitive disorder (MND), and HIV-associated dementia (HAD). These criteria are based on a comprehensive neuropsychological assessment that presupposes the availability of validated, demographically adjusted, and normative population data. Novel neuroimaging modalities and biomarkers have been proposed in order to complement NCI assessments, elucidate neuropathogenic mechanisms, and support HIV-associated NCI diagnosis, monitoring, and prognosis. By integrating neuropsychological assessments with biomarkers and neuroimaging into a holistic care approach, clinicians can enhance diagnostic accuracy, prognosis, and patient outcomes. This review interrogates the value of these modes of assessment and proposes a unified approach to NCI diagnosis.

## 1. Introduction

Neurocognitive impairment (NCI) can be a significant complication for individuals diagnosed with human immunodeficiency virus (HIV) infection/acquired immunodeficiency syndrome (AIDS), affecting various cognitive domains and sometimes leading to difficulties in the activities of daily living, even in people treated with combined antiretroviral treatment (cART) [1,2,3,4]. In the pre-cART era, NCI in patients with AIDS took the dramatic form of the AIDS dementia complex (ADC), a devastating, rapidly progressive cognitive decline that was also characterized by behavioral changes and motor dysfunction [5]. The annual incidence of ADC in patients with AIDS was 7%, whereas the overall life risk for a person with HIV infection was 5–20%. ADC had a poor prognosis, and, usually, survival did not exceed 6 months [6]. The introduction of cART in the mid-1990s led to a significant decrease in ADC cases and a shift towards milder forms of cognitive impairment [7,8]. However, treatment-naïve people living with HIV (PLWH) and those with incomplete viral suppression due to drug resistance or poor adherence remain vulnerable to NCI, whereas the burden for individuals under suppressive treatment is not yet clear [9]. In the modern cART era, NCI pathogenesis in PLWH is most commonly multifactorial, including legacy effects of HIV replication prior to cART initiation, low-grade chronic inflammation in the central nervous system (CNS), HIV persistence in the CNS compartment despite suppression in the periphery, cART neurotoxicity, and the cumulative effects of other comorbidities, especially for the ageing population with HIV infections [10,11]. A HIV-associated NCI diagnosis requires a multivariate approach, with neuropsychological (NP) assessments remaining the most important tool for diagnosing and categorizing the effects of HIV on the CNS [12]. Due to the inherent limitations of NP assessments, research has focused on biological indices to accompany and complement NCI investigations in PLWH [13]. Novel neuroimaging modalities and blood and cerebrospinal fluid (CSF) biomarkers have been extensively investigated to this end, but the incorporation of these tools in a clinical context has not yet been achieved [14,15,16].

This review aims to provide a comprehensive account of diagnostic approaches to NCI in PLWH, including the ‘gold standard’ NP assessment as well as the complementary biological and imaging biomarkers. Moreover, it attempts to map a way forward to the integration of these new diagnostic tools into a multi-faceted, holistic approach for PLWH with NCI.

## 2. Neuropsychological Assessments

### 2.1. Understanding Neurocognitive Impairment in People Living with HIV

Neuropsychological assessments are a well-established approach for the investigation of cognitive disorders. To be able to delve into the diagnostic methodologies, it is crucial to understand the nature of HIV-associated NCI. The common pattern of cognitive impairment, reported by numerous studies both before and after the cART era, has played a major role in guideline development for appropriate neuropsychological batteries [17,18,19,20]. NCI in PLWH is often referred to as having a “subcortical” pattern, with deficits predominately affecting executive functions, attention/working memory, speed of information processing, motor speed, episodic memory, and learning, while semantic memory, language, and visuospatial abilities are relatively preserved [12,18]. The onset is often insidious, with symptoms emerging gradually over time. Initially, individuals may experience subtle changes in cognition, such as forgetfulness, difficulties with concentration, or decreased processing speeds. As the disease progresses, more pronounced deficits in memory, language, and executive function become evident, impacting activities of daily living and social interactions [21].

According to Antinori et al., there is a range in the severity of HIV-associated neurocognitive disorder (HAND), varying from mild deficits to severe dementia [22]. In order to summarize the degree of severity, the group suggested a classification scheme known as the Frascati criteria (Table 1) to categorize patients in three diagnostic entities: asymptomatic neurocognitive impairment (ANI, with mild to moderate impairment in at least two cognitive domains without everyday life difficulties); mild neurocognitive disorder (MND, with mild to moderate impairment in at least two cognitive domains and mild everyday life difficulties), and HIV-associated dementia (HAD, with moderate to severe NP impairment in at least two cognitive domains and moderate to severe everyday life difficulties) [22]. In the presence of major depression or substance dependence, a HAND diagnosis is postponed until the remission of depression or the cessation of substance use. The severity of impairment across cognitive domains, as documented by the neuropsychological assessment, has been utilized as the gold standard for HAND diagnosis [22].

This classification has played a significant role over the years leading up to the present by informing guidelines for the neuropsychological battery for PLWH, resulting in studies attempting to provide a description of the cognitive phenotype in each of the diagnostic categories. For instance, ANI patients may present with mild deficits in memory, attention, speed of information processing, and motor speed and dexterity that remain unnoticed by the individuals. However, individuals with MND notice cognitive alterations (such as verbal fluency, working memory, etc.) and report them as symptoms, while individuals with HAD present severe cognitive deficits. Despite this categorization attempt, there are studies arguing that the pattern of cognitive impairment is inconsistent among individuals [18,23,24]. In addition, a recent longitudinal study focusing on the progression of NCI found that HAND might not advance invariably [25]. More specifically, it reported that approximately 70% of individuals with virologic suppression remained stable in a 4-year follow-up period, while some individuals even showed improvements. Nevertheless, the risk of progression remains, particularly for those initially categorized as having ANI [25].

The Diagnostic and Statistical Manual of Mental Disorders, version 5 (DSM-5) also provides a diagnostic schema for HAND by stratifying cognitive disorders into two categories based on the severity of symptoms: (a) mild neurocognitive disorders and (b) major neurocognitive disorders [26,27]. Mild neurocognitive disorders describe modest cognitive decline in ≥1 cognitive domains and no interference with the patient’s capacity for independence in daily living. A major neurocognitive disorder diagnosis requires a significant cognitive decline in ≥1 cognitive domains and at least some interference with the patient’s functionality of daily living [26,27]. A diagnosis of a major or mild neurocognitive disorder due to HIV infection is not made if a non-HIV condition or another mental disorder can better explain the cognitive decline [26,27]. The DSM-5 does not specify thresholds of impairment by identifying standard deviations below the average performance of a group with similar sociodemographic factors (which is usually the case). However, according to Tierney et al., performance is typically <1 SD in mild neurocognitive disorders and <2 SDs in major neurocognitive disorders [28]. Interestingly, in contrast to the Frascati criteria, the DSM-5 requires both objective (based on standardized neuropsychological assessments) and subjective (complaints) indications of impairment [28].

### 2.2. The Role of Neuropsychological Assessments

Neuropsychological assessments serve as a cornerstone in diagnosing NCI in PLWH due to their ability to detect subtle cognitive changes and provide a comprehensive profile of strengths and weaknesses [29,30]. These assessment involve a battery of standardized tests designed to evaluate various cognitive domains. Among the main goals of NP assessments in PLWH are: (a) to identify NCI directly associated with HIV, (b) to assess potential confounding comorbid conditions such as psychiatric illness, (c) to investigate potential links between HIV infection characteristics and the presence of NCI everyday functioning, (d) to determine cART effects, and (e) to provide clinicians and PLWH with feedback on the course and the outcomes [12]. Therefore, it is evident that neuropsychological assessments play a major role both in the diagnosis and the monitoring of HAND. 

According to the Frascati criteria, a complete NP evaluation should be able to assess, with at least two neuropsychological tests, the performance of at least five cognitive domains, including: language, memory and learning, attention/working memory, executive function, and motor dexterity [22]. To better understand the risk for HAND, Antinori et al. highlighted the importance of placing the neuropsychological assessment before the initiation of cART [22]. It was also suggested that the rationale of Woods et al. would be a good paradigm for the cognitive assessment of individuals with HIV [29]. More specifically, this study examined the interrater reliability of clinical ratings of neuropsychological impairment and neurocognitive diagnoses in individuals with advanced HIV infections [29]. The findings indicated excellent interrater reliability in rating the presence and severity of neuropsychological impairment but an overall modest reliability for neurocognitive diagnoses. Most diagnostic disagreements pertained to the etiology of impairments in individuals with medical and neuropsychiatric risk factors in addition to HIV infection [29].

While cognitive assessments are the cornerstone for an NCI diagnosis, it is equally vital to consider mood alterations, given the stressors that PLWH are facing [31]. Studies have reported a high prevalence of psychiatric comorbidities, including depression, anxiety, and posttraumatic stress disorder [32,33,34,35]. In a large North American cohort, the estimated prevalence of depression and anxiety in PLWH was 38.7% and 27.8%, 2.3 and 4.9-fold higher than that of the general population, respectively [32]. Depression has been linked with several adversities in the everyday life of PLWH, resulting in a worse quality of life and increased morbidity and mortality [36,37]. Depression is also associated with non-adherence to cART, higher rates of detectable viral load, and decreased functionality that might lead to reduced professional productivity and social isolation [38]. Furthermore, depression and anxiety have both been associated with the presence of HIV-associated NCI [39,40,41]. This association may be due to the effects of mood on cognitive processes, or it may underpin a common neuroinflammatory cause [42]. Other studies have tried to combine a set of psychological symptoms, including depression, anxiety, and distressing thoughts, into a latent factor, labelled as psychological distress, to more accurately assess subclinical or transdiagnostic symptomatology [43]. Psychological distress seems to be both a clinical outcome associated with HIV infection and a predictor of accelerated disease progression [43]. A recent study investigated the complex and bidirectional relationship between mental disorders and HIV considering sociodemographic and health-related factors. The results indicated that personalized interventions for psychological distress would be of great importance for PLWH, especially those of older age, females, those living in urban areas, and those with low or no education [44]. Neglecting to address depressive symptoms in NP assessments may lead to incomplete diagnoses and suboptimal management strategies. Therefore, integrating mood assessments alongside cognitive evaluations is crucial for an accurate diagnosis and a tailored treatment planning [45].

### 2.3. Challenges in Neuropsychological Assessments

Despite their utility, conducting neuropsychological assessments in PLWH presents several challenges. Some domains are assessed to a lesser extent than others due to limited tests used, focusing more on the effects of fronto-striatal and white matter involvement [12]. As a result, some deficits might be underdiagnosed by remaining untested. Another challenge pertains to the aforementioned variability in cognitive performance and patterns of dysfunction, which could be attributed to differences in HIV neuropathology, comorbid conditions, premorbid functioning, and medication side effects. These can confound test results, requiring careful consideration and interpretation by clinicians. The detection of the direct effects of HIV on the nervous system might be precluded by CNS opportunistic infections or prior illnesses or injuries. This is why determining the biological and environmental sources of these different patterns of impairment remains crucial [12]. 

However, one of the most significant challenges in neuropsychological testing is the lack of sensitivity and specificity provided by the neuropsychological tests available, which can lead to overdiagnosis of HAND based on the Frascati criteria [9,46]. In the CNS, based on the HIV Antiretroviral Therapy Effects Research (CHARTER) study, the prevalence of HAND was 52%, and 33% of the participants without comorbidities were classified as having ANI [1]. In addition, Gisslen et al. found that ANI classification has a high false positive rate, allowing them to characterize 16–21% of the general population as abnormal [46]. To accurately approach the prevalence of HAND, the Pharmacokinetic and Clinical Observations in People over Fifty (POPPY) study in the United Kingdom used three different definitions for HAND: the global deficit score (GDS), the multivariate normative comparison method, and the Frascati criteria. After adjusting the scores for the age and education of the subjects meeting the criteria for all three definitions, only 14% of the participants were found to be cognitively impaired [47].

A consensus on the use of specific tests for neuropsychological assessment in PLWH has not been reached [48]. The existing guidelines focus on the Frascati criteria, recommending a comprehensive NP evaluation to assess several cognitive domains and endorsing tests of preference for each cognitive domain [22]. The Mind Exchange Working Group recommends that neuropsychological tests undergo a validation procedure in the language and culture of the tested population, being scored according to the appropriate normative data [49]. However, there is significant variability in the tests available for assessing the same cognitive domains across different countries [50]. Moreover, test selection depends on several factors such as the availability of qualified personnel to administer and interpret the tests, the scope of the assessment, financial considerations, time availability, and the characteristics of the tested population in which the tool is intended for use [48]. 

While international organizations do not propose a specific NP test battery for HIV-associated NCI diagnoses, there are several recommendations regarding monitoring and screening. The commonly used screening tools are either HIV-specific, such as the HIV Dementia Scale (HDS) [51] and the International HIV Dementia Scale (IHDS) [52], or they are intended for use in the general population, e.g., the Mini-Mental State Examination (MMSE) and the Montreal Cognitive Assessment (MoCA) [53]. The Mind Exchange Working Group recommends the use of HDS or IHDS for the screening of all PLWH [49]; however, their psychometric properties have been brought into question due to their low accuracy [53,54]. In a systematic review, HDS had a 42% sensitivity and 91% specificity, while IHDS showed a 64% sensitivity and 66% specificity for diagnosing HAND [48,54]. The MMSE and MoCA have also been found to be unreliable as screening tools in PLWH, although the latter has shown better sensitivity for milder NCI [55,56]. In conclusion, a reliable screening tool with an acceptable sensitivity and specificity for HIV-associated NCI in the cART era is currently lacking.

### 2.4. New Approaches to HIV-Associated NCI Definitions

**Figure 1:**
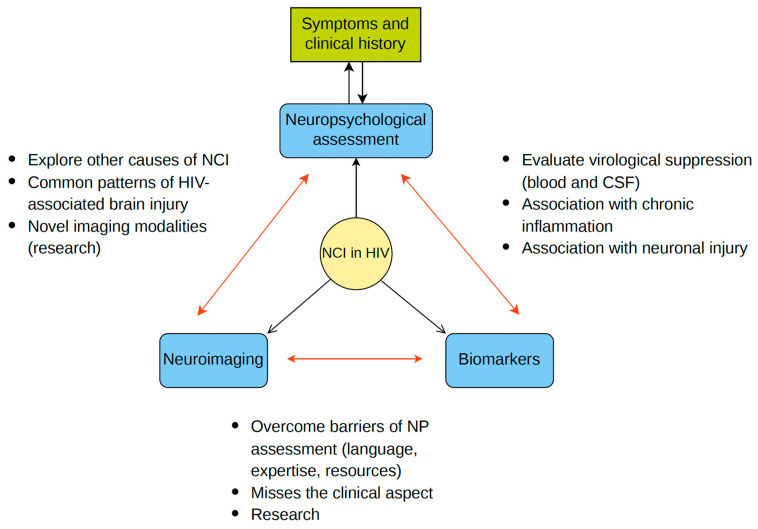
Multimodal assessment of neurocognitive impairment (NCI) in people living with HIV- NCI is primarily focused on performance on neuropsychological (NP) assessments. NP evaluation is informed by the individual’s symptoms and clinical history, allowing for a comprehensive investigation of the potential contributing and confounding factors of NCI. This is a bidirectional relationship, as the NP assessment may also provide evidence for the presence of alternative causes of NCI other than HIV. Neuroimaging can help to identify an underlying cause of the impairment, as well as to provide evidence to support an HIV-associated brain injury diagnosis. On the other hand, biomarkers of inflammation and neuronal injury, albeit not standardized, may show evidence of neuropathology and can be used for monitoring purposes. CSF sampling is useful in order to identify HIV CSF escape, opportunistic infections and malignancies, neurosyphilis, and other inflammatory processes such as immune reconstitution inflammatory syndrome (IRIS). When an NP assessment is not feasible or accessible, neuroimaging in combination with CSF investigations may help to identify patterns of CNS disease that explain the clinical symptoms.

Over the years, the Frascati criteria have been criticized for high rates of false positive results, the inherent weaknesses of normative data used for deficit calculations, and the fact that for the ageing population with HIV, NCI is usually multifactorial and cannot be directly attributed to HIV neuropathogenesis [11,46,57]. Also, the classification of asymptomatic individuals with a low performance on cognitive tests as neurocognitively impaired does not take into account social determinants of health, premorbid functioning, and comorbidities that may differ in PLWH [57]. Nightingale et al. recently presented a new consensus to guide NCI diagnosis in the future, stressing the importance of the clinical context when assessing cognitive function in PLWH [11]. The group recommends abandoning the term ANI in favor of ‘low performance on cognitive test’ and coins a new term for NCI that is directly attributed to HIV, namely HIV-associated brain injury (HABI). HABI in turn is separated into legacy (brain injury from pretreatment damage) and active categories. Active HABI represents ongoing damage resulting in clinical and/or radiological progression. Supporting evidence of damage via clinical history and examination and neurological investigations including neuroimaging and CSF sampling are required to accurately assess the presence of HABI (Table 2, Figure 1) [11].

## 3. Neuroimaging in HIV-Associated NCI

Neuroimaging serves as a valuable tool in the diagnosis and management of HIV-associated NCI as well as its differential diagnosis from other causes of NCI such as Alzheimer’s disease and vascular dementia. Over the last decades, a wide range of innovative, non-invasive neuroimaging techniques has been developed and applied in the investigation of NCI in PLWH (Table 3). Regarding magnetic resonance imaging (MRI), three main approaches are employed: metabolic (magnetic resonance spectroscopy—MRS), structural (MRI volumetrics and diffusion tensor imaging—DTI), and functional (functional MRI—fMRI) [58]. Positron emission tomography (PET) has also been a valuable tool to assess metabolic brain parameters, such as neuroinflammation, neuronal function, microglial activation, and neurotransmitter signaling [59].

### 3.1. Proton Magnetic Resonance Spectroscopy

While MRI detects the magnetic resonance signal of hydrogen atoms in water and brain lipids, the technique can be modified to identify the levels of important cell metabolites such as N-acetyl-aspartate (NAA), a widely used marker for neuronal integrity [60]. Other brain metabolites include creatine (Cr), involved in cellular energy metabolism, choline, indicative of cell membrane and neurotransmission metabolism, myo-inositol (MI), related to the density and activation of neuroglial cells, glutamic acid/glutamine (Glx), an excitatory neurotransmitter increased in excitotoxicity, and gamma-aminobutyric acid (GABA), a key inhibitory neurotransmitter [86]. Due to methodological constraints in quantifying these metabolites, their ratios to Cr are often used [58]. However, in the case of HIV infection, using Cr as a reference has been deemed precarious, as Cr levels correlate with HIV RNA and cannot be used for normalization of other metabolites [87,88]. To address this issue, the use of water as an internal reference marker has been proposed [86].

The classical metabolic pattern found in HIV infection involves a decrease in NAA and Glx, indicating neuronal loss, damage, or dysfunction alongside an increase in choline and MI that is attributed to inflammation and activated microglia [86,89,94]. In early infection, an initial rise in choline and MI is observed, followed by a decrease in NAA, signaling neuronal integrity loss. These findings, particularly in the frontal white matter and basal ganglia, appear to correlate with the severity of neurocognitive deficits [66,88,89] as well as with the levels of peripheral inflammation biomarkers such as monocyte chemoattractant protein-1 (MCP-1) and soluble CD14 (sCD14) [103]. In individuals on cART, a mitigation of these metabolic disturbances is observed, correlating with an increase in CD4+ lymphocyte count and improved performance in neuropsychological tests [90,104]. However, some studies suggest that these disturbances persist despite cART use in the context of chronic and stable HIV infections [91,105].

### 3.2. MRI Volumetrics

For the identification of structural brain changes, structural MRI is typically used, with image processing software used for morphometric analysis. Common findings in morphometric studies of the brain in PLWH include brain atrophy, both diffuse and localized, in specific areas of gray and white matter [106]. Volume loss often involves subcortical structures such as the caudate nucleus, lentiform nucleus, amygdala, hippocampus, and thalamus, as well as segments of white matter including the corpus callosum and the radiate crown [107].

During the cART era, HIV infection is characterized by atrophy in both subcortical and cortical gray matter, revealing brain volume loss despite antiretroviral treatment [108]. This atrophy is more pronounced in advanced stages of infection, but changes are observed even in PLWH without cognitive impairment [109]. Structural brain changes appear to commence early in HIV infection and may manifest as cortical atrophy and enlargement of the third ventricle [110]; however, these findings were not confirmed in a recent study involving a large number of patients [111]. In the same study, it was revealed that a longer duration of untreated HIV infection is associated with volume loss in the thalamus, caudate nucleus, and parahippocampal gyrus as well as thinning of the cortex in the frontal and parietal lobes and the cingulate gyrus. After the initiation of cART, no further volume loss was observed; in fact, there was a small but significant increase in cortical thickness in the frontal and temporal lobes, which requires further investigation [111]. The volumetric changes in the brain are also associated with neurocognitive performance, HIV viral load, and the nadir of CD4+ lymphocytes [58].

### 3.3. Diffusion Tensor Imaging

Diffusion tensor imaging (DTI) is used for the assessment of white matter integrity in vivo, utilizing the diffusion of water molecules through brain tissue and allowing for visualization, analysis, and quantification of major neural pathways [58]. Water diffusion in the brain is restricted along the axons due to the presence of the myelin sheath. DTI takes advantage of this principle to map the connectivity between different brain regions and the integrity of axons [60]. Four diffusion parameters are generated by DTI: (a) fractional anisotropy (FA), related to the coherence of white matter neuronal pathways, (b) mean diffusivity (MD), which represents the sum of water molecule diffusion in space, (c) axial diffusivity (AD), expressing the diffusion along the neuron, and d) radial diffusivity (RD), indicating diffusion perpendicular to the longitudinal axis [112].

Changes observed in HIV infection using DTI primarily involve reduced fractional anisotropy in the internal capsule and sections of the frontal, corona radiata, and splenium white matter, along with increased mean diffusivity in the corpus callosum and white matter tracts such as the cingulum, fornix, and anterior thalamic radiation [106]. The lower nadir of CD4+ lymphocytes has been correlated with diffuse increases in mean diffusivity in the white matter of both hemispheres and the corpus callosum, accompanied by disturbances in information processing speed and motor skills [113]. It has also been suggested that HIV infection, in combination with age, contributes to white matter integrity loss, leading to accelerated brain aging [70].

### 3.4. Functional Magnetic Resonance Imaging

Functional MRI, unlike other modalities used for evaluating anatomical integrity, is used for the in vivo assessment of brain metabolic activity [107,114]. The blood oxygen level dependent (BOLD) method that is commonly used in fMRI detects the momentary increase in blood and oxygen flow in activated brain areas [58,115]. Essentially, fMRI-BOLD measures the coupling degree between neural activity and blood flow in the region of the brain that is activated during a specific task being performed by the subject or during rest through changes in paramagnetic deoxyhemoglobin concentrations [115]. fMRI can be performed with the subject either in a resting state or being subjected to an experimental condition. The use of fMRI in a resting state (resting-state fMRI—rs-fMRI) allows for the evaluation of spontaneous BOLD fluctuations, detecting an intrinsic property of the brain, functional segregation, or specialization of brain areas and networks [13,116]. The most significant resting-state networks of the brain include the default mode network (DMN), associated with internal focus, mind wandering, and recall, the executive control network (ECN), involved in performing demanding cognitive functions such as decision-making and working memory, the salience network (SAL), which detects salient internal or external stimuli and mediates the DMN and ECN, and the visual, sensory–motor, and attention networks. In contrast, task-based fMRI detects functional changes occurring in an individual’s brain during the performance of a cognitive task (e.g., observing a visual or auditory stimulus, processing linguistic content, decision-making processes) [60]. For the interpretation of fMRI results, two measures are mainly used: regional homogeneity and functional connectivity [13].

Studies using rs-fMRI in HIV infection have shown reduced connectivity within the visual network, decreased functional connectivity in the splenium pathway, and decreased synchronization in the SAL and ECN networks [13,82,84]. Reduced functional connectivity has also been observed between the fusiform gyrus and prefrontal cortex [77]. Regarding task-based fMRI, individuals with HIV infection exhibit increased BOLD activity in the occipital lobes during the performance of a simple attention task as well as in the frontal and occipital lobes during more complex attention tasks [117]. Additionally, increased activation in left occipital and bilateral frontal regions was observed during a decision-making task with economic content [118]. These changes in PLWH may reflect a mechanism of compensating for their cognitive deficits through increased activation or “recruitment” of additional brain regions compared to individuals without HIV infection [83].

The data from longitudinal fMRI studies are sparce and not homogenous. Zhuang et al. showed that treatment-naïve PLWH had significantly lower functional connectivity compared to a matched control group, but they improved after 12 weeks of cART [119]. In a recent longitudinal study of cART intensification, in HIV-associated NCI, an increase in functional connectivity was found in major resting-state networks after 12 months [80]. Functional connectivity changes have also been found in people switching cART, suggesting that fMRI could be used for investigating cART neurotoxicity [120]. fMRI has been proposed as a reliable imaging biomarker for NCI diagnosis and monitoring, as it differentiates both individuals with and without HIV-associated NCI and those with and without HIV infection. However, some studies have failed to identify differences in functional connectivity between these groups [85,121].

### 3.5. PET

Neuroinflammation and neuronal function during the course of HIV infection can be assessed through glucose metabolism visualization using 18F-fluorodeoxyglucose (FDG) PET [59]. Neuroinflammation is evident in untreated HIV infection by increased uptake of the ligand in the subcortical areas of the brain. Chronic and treated HIV infections result in hypometabolism on 18F-FDG PET, possibly related to neuronal loss [102]. In longitudinal studies, cART initiation has been associated with an increase in the ligand uptake in the frontal cortex, suggesting a return-to-normal effect, while hypometabolism persists in subcortical structures [96]. However, a recent study that compared men who have sex with men (MSM) living with HIV and MSM using pre-exposure prophylaxis (without HIV infection) did not find differences in brain metabolism using 18F-FDG PET, but it showed that recreational drug use was associated with prefrontal cortex hypometabolism in both groups [122].

In recent years, 18-kDa translocator protein (TSPO) PET has been employed in order to detect microglia activation in PLWH. Findings from TSPO PET studies are generally conflicting due to the use of different radiotracers, differences in NCI definition, small sample sizes, methodological variability, and study design [16]. A study that compared PLWH on cART with HIV-negative controls found globally increased radiotracer uptake, especially in the subcortical grey matter. Increased uptake was associated with worse performance in verbal learning and memory tasks, a lower CD4/CD8 ratio, higher pre-cART HIV-RNA, and increased markers of microbial translocation [101].

### 3.6. Role of Neuroimaging in Differentiating HIV-Associated NCI from Other Causes of Cognitive Decline

Differential diagnosis between cognitive decline attributed to HIV infection and other causes of NCI, particularly Alzheimer’s disease (AD) and vascular dementia, is of great importance due to the increase in the PLWH population over the age of 60 [123]. Neuroimaging can play a significant role to this end, identifying the characteristic patterns that each type of neurogenerative disease has. However, HIV and non-HIV etiologies may often coincide and pose a diagnostic challenge.

HIV-associated NCI is traditionally described as a ‘subcortical’ disease, primarily leading to volume reductions in the basal ganglia, thalamus, cerebellum, and frontal white matter, whereas AD is characterized by a ‘cortical’ pattern of atrophy, affecting the mesial temporal lobe and temporoparietal cortex [124,125]. Beyond the macrostructural differences detected using computed tomography (CT), or more accurately using MRI, DTI can additionally reveal microstructural differences in white matter integrity. HIV infection commonly causes a widespread pattern of white matter abnormalities with a frontal predominance; in contrast, AD is associated with changes that are more evident in the posterior white matter and fornix [126,127]. Another powerful tool used to differentiate between HIV-associated NCI and AD is PET. Amyloid and tau PET is considered to be a sensitive and specific neuroimaging biomarker for AD, with recent criteria proposing that amyloid- and tau-positive PET suffice for an AD diagnosis [128,129]. This is different from HIV-associated NCI, which is not associated with increased amyloid binding [130,131]. Vera et al. recently showed that amyloid PET imaging significantly contributed to the differential diagnosis of NCI in PLWH, increasing the diagnostic certainty and influencing patient management [132]. MRI is the recommended neuroimaging technique for evaluating the presence of vascular cognitive impairment, but when it is unavailable, a CT may provide evidence of atrophy and vascular lesions [133]. Vascular dementia is expected to be more prevalent in the ageing cohort of PLWH due to the cumulative effects of traditional risk factors as well as HIV and cART effects on endothelial function [134]. Although vascular cognitive impairment (VCI) caused by large vessel disease, namely infarcts, can be easily visualized using standard neuroimaging, for cerebral small vessel disease (CSVD) that often diffusely involve the white matter, DTI and multi-modal imaging may be required to differentiate it from HIV-related lesions [135]. Furthermore, there is growing evidence to suggest that CSVD shares some common pathways with HIV neuropathogenesis, including chronic inflammation and BBB dysfunction [136]. More studies are needed to investigate the interaction between CSVD and HIV-associated NCI, utilizing advanced imaging modalities and a longitudinal design.

## 4. Diagnostic and Prognostic Blood and CSF Biomarkers

A biomarker is defined as “a measurable characteristic that objectively evaluates and assesses physiological, pathogenetic processes, or pharmacological responses to a therapeutic intervention” [137]. The ongoing proliferation of HIV in the CNS results in increased levels of soluble and cellular biomarkers, which, while diminishing after cART initiation, usually do not return to normal levels, despite viral suppression [138,139]. However, in individuals starting therapy during acute HIV infection, almost normal levels of biomarkers in the CSF—but not in plasma—may be reached [140]. Soluble biomarkers in plasma and CSF are usually expressed by specific cell types and can provide information about the underlying pathogenetic mechanisms of CNS injury and HAND. However, the specific molecular pathways controlling the expression of these biomarkers are still under investigation [139].

The value of various plasma and CSF biomarkers has been investigated for both symptomatic and subclinical/asymptomatic neurocognitive impairment. Neuronal injury and local immune activation indicators are among the most promising biomarkers in the CSF [141]. For example, neopterin in CSF, a biomarker of macrophage activation, increases during HIV infection and reaches higher levels in HAD. Additionally, neopterin and neurofilament light chains (NFL) are found at higher levels in the CSF of individuals with HIV and mild neurocognitive impairment compared to those without deficits [142]. Biomarkers related to inflammation and immune activation, which are associated with both monocyte/macrophage and lymphocyte activation, have been studied. For instance, soluble CD14 (sCD14) and CD163 (sCD163) macrophage receptors are associated with HIV-related neuronal injury, while activated CD14+CD16+ monocytes correlate with overall cognitive function, executive functions, speed of information processing, and 12-month incidence of NCI [143]. On the other hand, since the entry of T lymphocytes into the CNS is generally restricted, markers of lymphocyte activation are mainly associated with acute HIV infection, CD8+ T encephalitis, HAD, and HIV CSF escape.

### 4.1. Monocyte/Macrophage Activation Markers

#### 4.1.1. Neopterin

Neopterin is the metabolic byproduct of triphosphoric guanosine breakdown and is an indicator of cellular immune activation. It is mainly produced by monocytes/macrophages and dendritic cells in response to interferon production, especially interferon-gamma (IFNγ), a T helper (Th)1 cytokine [144,145]. CSF neopterin levels remain elevated in individuals not receiving cART and are inversely related to CD4+ T cell count and CD4+/CD8+ T cell ratio, while they appear to decrease after treatment initiation [144,146]. Furthermore, neopterin may be pathophysiologically linked to neurodegeneration in the CNS, as it is associated with the release of reactive oxygen species (ROS), nuclear factor kappa B (NF-κB), cytokines, and inflammatory mediators as well as intercellular adhesion molecule-1 (ICAM-1) [144].

CSF neopterin levels, while not specific to HAND, correspond to the severity of NCI in PLWH, while elevated levels also are of prognostic value, being correlated with up to a 7 times greater risk of HAD and HIV-related mortality [147,148,149]. Moreover, increased plasma neopterin levels are associated with reduced hippocampal volume as well as poorer performance in tests of semantic memory, working memory, and overall cognitive function [150].

#### 4.1.2. sCD14, sCD163, and LPS

sCD14 is the soluble form of the monocyte receptor for microbial lipopolysaccharides (LPSs) and is released from the cell membrane upon cell activation [151]. LPS increases in plasma due to the transportation of microbial products through the intestinal mucosa into the systemic circulation, which has been associated with monocyte activation and increased migration of infected cells to the brain [145]. Due to technical difficulties in reliably measuring LPSs in plasma, sCD14 is often used as a surrogate marker. Both sCD14 and LPSs are associated with attention and learning, while sCD14 is also correlated with the speed of information processing [152,153]. Moreover, elevated plasma and CSF sCD14 levels are associated with worse overall cognitive performance [154,155,156].

CD163 is a scavenger receptor for the haptoglobin–hemoglobin complex, mediating haptoglobin clearance by macrophages [157]. sCD163 results from proteolytic cleavage on the cell surface and increases in various pro-inflammatory conditions, and it is an indicator of monocyte/macrophage activation and expansion [158]. In the context of HIV infection, sCD163 remains elevated despite cART and is associated with persistent monocyte activation and inflammation in the CNS. Research findings regarding the value of sCD163 as a potential biomarker for diagnosing and monitoring HAND are not conclusive [145,159]. Studies suggest a connection between elevated plasma and CSF sCD163 levels and low cognitive performance [160].

#### 4.1.3. MCP-1/CCL2

Monocyte chemoattractant protein-1 (MCP-1) or CCL2 is a potent inducer of monocyte chemotaxis and migration across the blood–brain barrier (BBB) into the CNS. HIV-infected monocytes entering the CNS infect and activate other brain-resident cells such as perivascular macrophages, microglial cells, and astrocytes [92,156,161]. MCP-1 production in the CNS is induced by the HIV Tat protein and tumor necrosis factor alpha (TNFα), and it is associated with HAD diagnosis and prognosis [162,163,164]. In individuals with long-standing HIV infection on treatment, MCP-1 levels in plasma correlate with diffuse fractional anisotropy reduction and mean diffusivity increases in the midbrain, corona radiata, and superior longitudinal fasciculus on DTI, indicating ongoing chronic inflammation and white matter disruption [73].

#### 4.1.4. CD16+ Monocytes

In healthy individuals, the majority of circulating monocytes express the CD14 receptor on their surface, while <10% exhibit an activated, intensely pro-inflammatory phenotype characterized by the expression of CD16 [139]. In individuals with HIV, the percentage of CD16+ monocytes can reach up to 40% of circulating monocytes, especially in those with NCI [165]. CD16+ monocytes, stimulated by MCP-1, more effectively cross the BBB due to the expression of cell migration molecules (CXCR5, CX3CR1, and integrin CD11b) and are implicated in the pathogenesis of HIV-associated NCI [165,166]. A higher percentage of CD16+ monocytes is associated with worse overall cognitive performance, executive function, and speed of information processing, serving as a prognostic factor for neurocognitive decline 12 months later [143].

### 4.2. Biomarkers of Neuronal Damage

#### 4.2.1. Neurofilaments

Within the CNS, neurofilament proteins, belonging to the intermediate filament class, are heteropolymers composed of four subunits: neurofilament heavy (NFH), neurofilament medium (NFM), neurofilament light (NFL), and α-internexin [167]. NFL is a major structural component of neuronal fibers, contributing to shape maintenance, axon growth, and efficient electrical signal propagation [145]. After neuronal and axonal damage, NFL levels increase in the CSF as they are released from injured neurons. Elevated NFL levels have been observed in various neurodegenerative diseases such as Alzheimer’s disease, multiple sclerosis, and amyotrophic lateral sclerosis [168].

During HIV infection, CSF NFL levels increase in response to HIV-related neuroinflammation, decrease with cART initiation, and rebound after treatment interruption [169]. They are positively correlated with plasma HIV-RNA and inversely correlated with CD4+ T cell count. The contribution of monocyte/macrophage activation in neurological damage becomes evident based on the direct correlation of NFL with sCD14 and sCD163 [170]. To overcome the limitations that accompany using a lumbar puncture for CSF sampling, a relatively novel, highly sensitive method has been developed for NFL measurements in plasma, showing a high correlation with CSF levels [171,172].

NFLs, both in the CSF and plasma, are considered to be promising biomarkers for HIV-associated NCI diagnosis and monitoring [173]. In individuals not receiving cART, the concentration of NFLs in the CSF is strongly and independently correlated with CSF neopterin concentration and white blood cell count [174]. Moreover, BBB disruption, as assessed based on the CSF/plasma albumin ratio, has been associated with increased NFL levels, the presence of neurological injury, and intrathecal immune activation in neuro-asymptomatic individuals [175]. After cART initiation, NFLs decline and reach age-appropriate levels within a few months, highlighting the importance of promptly starting antiretroviral treatment to prevent further neurological injury [174,176].

#### 4.2.2. S100 Calcium-Binding Protein B (S100B)

The S100B protein is produced by astrocytes and oligodendrocytes and is detected in high levels in the CNS or plasma in case of a neuronal injury. For this reason, it is considered to be a peripheral indicator of CNS damage, BBB disruption, and specifically astrocyte activation and reactive gliosis [145]. The physiological function of S100B depends on its concentration in the extracellular fluid. At low levels, it promotes neuronal survival and regulates intracellular cooperation, while at high levels, it leads to neuronal death and the production of proinflammatory cytokines [177]. The diagnostic and prognostic value of measuring S100B levels, both in the CSF and plasma, has been questioned, as different studies present conflicting findings, with some associating increased levels with symptomatic NCI and others reporting the absence of deficits [178,179,180,181,182]. It has been suggested that, as an indicator of active CNS injury, S100B may increase in the early stages of HAND and decrease in advanced but non-progressing HAND [168]. Therefore, the correlation between S100B levels and HIV-associated NCI remains uncertain, and more studies are needed to clarify its role as a biomarker of neurocognitive decline [182].

### 4.3. Biomarkers of Inflammation

Based on the premise that neuropathogenesis in PLWH is linked with persistent inflammation, researchers have sought to uncover potential correlations between specific plasma and CSF inflammation biomarkers and NCI [183,184]. The current understanding is that while cART initiation is associated with reduced levels of inflammation markers, these do not reach normal levels, as a low-grade chronic inflammation persists and contributes to HIV-associated comorbidities [185]. Biomarker measurement in the CSF has been the most promising due to the CSF acting as a proxy for CNS, while plasma biomarkers, albeit more convenient, are considered to be less specific. CSF and plasma pro-inflammatory cytokines and chemokines such as TNFα, IFNγ, ΙL-2, and IL-6 are elevated during primary HIV infection but decline over the first few months, even without cART initiation [186]. Studies assessing the correlation between inflammation biomarkers and NP performance have shown conflicting results [145]. Chemokines IP-10/CXCL10 and RANTES/CCL5 in plasma have been found to correlate with and strongly predict NP performance, as measured by the GDS, in treatment-naïve PLWH [187,188]. Moreover, CSF TNFα levels remain increased even in PLWH on long-duration cART compared to matched controls [20]. Whether this pro-inflammatory milieu is directly involved in HIV neuropathogenesis or it is mediated by other comorbidities is an area of active research, with studies identifying a complex immune interplay between HIV infection and metabolic abnormalities, substance use, and even depression [42,189,190].

### 4.4. Extracellular Vesicles

Extracellular vesicles (EVs), a means of intercellular communication and regulation, have gained attention in recent years as a promising biomarker for various neuroinflammatory and neurodegenerative states including HIV-associated NCI [191,192]. EVs, categorized according to their size into exosomes (30–150 nm) and microvesicles (150–1000 nm), carry and deliver biologically active molecules such as lipids, nucleic acids, and proteins as well as pathogenic substances including viral proteins [192]. In a study that measured CSF EVs, Guha et al. found higher levels in PLWH compared to people without HIV infection and in PLWH with NCI versus no NCI [180]. A proteomic analysis revealed a neuronal, glial, and choroid plexus origin of these EVs, expressing an astrocytic, pro-inflammatory, and stress-associated phenotype [193]. Another study found that plasma EVs derived from activated monocytes, as well as plasma and CSF EVs that expressed neuronal markers, were associated with HIV-associated NCI [194]. Furthermore, neuron-derived exosomes in the plasma of PLWH with NCI are enriched with markers of neuronal damage such NFLs, high-mobility group box 1 (HMGB1), and amyloid beta [195,196]. Research has also focused on EVs containing the HIV protein Nef, as they have been found to exert multiple deleterious effects, both in the periphery and in the CNS, and have been associated with HIV neuropathogenesis [192,197]. However, the value of Nef EVs as a biomarker for HIV-associated NCI has yet to be shown.

## 5. Combining Neuropsychology, Imaging, and Biomarkers in HIV-NCI Diagnosis

Despite its limitations, neuropsychological assessment remains the mainstay for NCI investigation in PLWH. A detailed clinical history, mental health assessment, and lifestyle information are essential in order to establish a differential diagnosis and investigate causes not related to HIV [198]. Mental health problems such as depression and anxiety, bipolar disorder, and substance-induced mood disorder disproportionately affect PLWH, and their presence may significantly confound cognitive symptoms and neurocognitive performance [11]. Both the Frascati criteria and the DSM-5 preclude an HIV-associated NCI diagnosis in the presence of a major mental disorder. Therefore, mental health problems need to be carefully screened for, diagnosed, and treated before an HIV-associated NCI diagnosis is made [199].

According to Nightingale et al., a combination of investigations and biomarkers may be used to diagnose HIV-associated brain injury (HABI) in order to provide sufficient evidence of direct neuronal damage caused by HIV [11]. This is a departure from the Frascati criteria, which are primarily based on strictly defined neuropsychological criteria [22]. Despite research advances, a standardized way of incorporating neuroimaging and biomarkers into the investigation for NCI in PLWH is yet to be established [139]. Furthermore, these investigations may not be routinely available in resource-poor settings. These drawbacks should be taken into consideration when establishing research and diagnostic criteria for HIV-associated NCI.

For research purposes, Nightingale et al. propose a combination of at least two out of three components in order to diagnose cognitive impairment, namely cognitive symptoms, low cognitive performance, and evidence of brain injury based on neurological investigations. In this context, neurological investigations include neuroimaging, CSF sampling, and appropriate biomarkers [11]. The integration of evidence of multiple sources is warranted, since there still is no single imaging or biomarker that can be used for HABI diagnosis. This classification avoids making an NCI diagnosis in people with low cognitive performance but no evidence of symptomatic decline or brain pathology, and it bypasses the problem of the false-classification rate that is inherent to neuropsychological assessments [57]. Therefore, the standardization of these investigations for clinical and research purposes is imperative. The European AIDS Clinical Society (EACS) guidelines have recently incorporated this approach when evaluating PLWH with cognitive symptoms, calling for a combination of NP assessment, neurological examination, brain MRI, and CSF examination after the exclusion of obvious confounding conditions [200].

When investigating symptomatic cognitive decline in PLWH, brain imaging is of paramount importance in order to exclude other causes of impairment and establish brain pathology. Novel neuroimaging approaches can help to elucidate structural and functional pathways of neuronal damage in the different stages of HIV infection [201]. Additionally, serial imaging may capture both an ongoing brain injury that could be amenable to treatment and the effects of cART initiation or switching [80,120]. However, imaging alone is neither sensitive nor specific for diagnosing NCI, while novel modalities such as fMRI and PET have been used only for research purposes, and so far, no thresholds for normality have been identified. Additionally, evidence of neuroinflammation in imaging does not necessarily translate into clinical disease, and it is not yet clear whether this represents ongoing injury or a healing process [202]. Further research is needed in order to optimally incorporate the advantages of these investigations in clinical practice.

Plasma and CSF biomarkers have been attractive targets as indirect measures of brain injury and inflammation [183,203]. Identifying a sensitive and specific set of biomarkers to diagnose HIV-associated NCI, as is the case for Alzheimer’s disease, has not yet been possible. NFLs have been the most promising biomarker for this purpose, and they have been systematically found to correlate with cognitive performance, especially in treatment-naïve PLWH [169,171,173,176]. Moreover, normal ranges in CSF and plasma per age group have been established [204]. Although not specific for HABI, high NFL levels, in association with a relevant clinical context, may complement HABI diagnoses, and serial measurements could be used for assessing the response to treatment. However, their use in routine clinical practice is not currently recommended. On the other hand, CSF sampling is essential to exclude CSF escape as the cause of neurocognitive symptoms as well as other causes of NCI such as neurosyphilis and opportunistic CNS infections.

## 6. Conclusions

The field of NCI in PLWH is undergoing an immense transformation. This has been guided by the widespread use of cART, which has strikingly improved the prognosis of HIV infection. Additionally, advances in neuroimaging and biomarker measurements have improved our understanding of HIV neuropathogenesis. The diagnostic approach to neurocognitive disorders in HIV heavily relies on neuropsychological assessments, which offer a sensitive and comprehensive means of identifying cognitive deficits. Challenges such as the presence of comorbidities and mental health problems, the psychometric competence of the neuropsychological tests used, and a tendency for NCI overdiagnosis or underdiagnosis highlight the need for a multimodal diagnostic approach. By integrating neuropsychological assessments with biomarkers, neuroimaging, and a holistic care approach, clinicians can enhance diagnostic accuracy, prognosis, and patient outcomes.

## Figures and Tables

**Table 1:** Frascati criteria for HAND diagnosis [22].

HAND Category	NP Criteria	Functioning
ANI	≥1 SD below adjusted norms in ≥2 cognitive domains	No impairment
MND	≥1 SD below adjusted norms in ≥2 cognitive domains	Mild impairment in everyday functioning
HAD	≥2 SD below adjusted norms in ≥2 cognitive domains	Severe impairment in everyday functioning
NP evaluation: assessment of ≥5 domains, preferably by two tests each, including: attention/working memory, language/verbal fluency, abstraction/executive skills, motor skills, memory (learning/recall), visuospatial perception, speed of information processing		
Functioning: self-report, proxy report from a significant other or caregiver, performance-based assessment of everyday functioning [17]		
Exclusion criteria: delirium, psychiatric illness, psychoactive substance use, alcohol use disorder, opportunistic CNS infections		

Abbreviations: ANI, asymptomatic neurocognitive impairment; CNS, central nervous system; HAD, HIV-associated dementia; HAND, HIV-associated neurocognitive disorder; MND, mild neurocognitive disorder; NP, neuropsychological; SD, standard deviation.

**Table 2:** Differences between the Frascati criteria (Antinori et al. [22]) and recommendations by the International HIV-Cognition Working Group (Nightingale et al. [11]).

	Frascati Criteria (HAND) (2007) [22]	Nightingale et al. (2023) [11]
Purpose	Research	Research and clinical
NCI classification	Not impaired, ANI, MND, HAD. Based on neuropsychological criteria and exclusion of confounding conditions.	ANI is changed to ‘low cognitive performance’ and is not considered NCI per se. Clinical history and/or neurological investigations in addition to low cognitive performance are required for an NCI diagnosis.
Symptom definition	Interference with daily functioning.	Changes in cognition, self-reported or by proxy, independently of daily functioning.
Pathogenesis attributed to HIV	In relation to incidental, contributing, and confounding factors. Presence of a confounding condition precludes HAND diagnosis.	Defines HIV-associated brain injury (HABI) for direct HIV neuropathogenesis. HABI is defined in relation to HIV suppression. HABI in individuals with plasma HIV-RNA suppression may be legacy (pretreatment) or active.
NP data interpretation	Based on quantitative deviation from normative NP data.	Favors use of NP performance as a continuous variable, assessed longitudinally, rather than a binary cut-off. False-classification rate of NP battery should be considered.

Abbreviations: ANI, asymptomatic neurocognitive impairment; HABI, HIV-associated brain injury; HAD, HIV-associated dementia; HAND, HIV-associated neurocognitive disorder; HIV-RNA, human immunodeficiency virus ribonucleic acid; MND, mild neurocognitive disorder; NCI, neurocognitive impairment; NP, neuropsychological.

**Table 3:** Findings and correlates of neuroimaging modalities in people living with HIV.

Imaging Modality	Findings	Clinical/Biological Correlates	References
MRI			[60,61,62,63,64,65,66,67]
Morphometry/Volumetry	Reduced size of basal ganglia, caudate nucleus, corpus callosum, hippocampus Reduced size in sensorimotor and supplementary motor cortex expansion of lateral ventricles	Correlates with NP performance, especially MND and HAD GM reduction correlates with nadir CD4+ hippocampal and thalamic volumes correlate with current CD4+ Regional atrophy correlates with HIV RNA, CSF HIV RNA, PBMC HIV DNA	
DTI	Lower FA, higher MD (especially WM: subcortex, corpus callosum [splenium and/or genu], centrum hemiovale)	Correlation with severity of dementia and specific cognitive domains, apathy, differentiation from aging brain Correlation with BBB disruption Correlation with plasma VEGF, MIP-1α, MIP-1β, MCP-1, sCD14	[68,69,70,71,72,73,74,75,76]
fMRI			[77,78,79,80,81,82,83,84,85]
task-based	Reduced activity of attention networks and Increased recruitment of adjacent areas to meet attention task demands Reduced cerebral blood flow during visual tasks	Differentiation HAND vs. non-HAND vs. seronegative, correlation with NP assessment	
resting-state	Decreased FC in cortico-striatal networks, precuneus, prefrontal region Decreased FC in executive, salient, and default mode networks	Improves with cART initiation	
MRS	Increased choline and MI in frontal WM and basal ganglia due to neuroinflammation and microglia activation (early) Reduced NAA due to loss of neuron integrity (later stages) Reduced Glx due to excitotoxicity	Correlation with NP assessment Correlation with MCP-1, sCD14, IP-10 Improvement with cART initiation, CD4+ Increase is associated with improvement of MRS findings	[86,87,88,89,90,91,92,93,94]
PET			[95,96,97,98,99,100,101,102]
FDG-PET	Increased glucose uptake in basal ganglia and thalamus, reversed with cART, eventually hypometabolism (legacy effect)	FDG PET improves with cART initiation, overlaps with CVD effects	
TSPO PET	Conflicting results: higher binding of ligand in diverse brain regions, others found no differences between HIV+ and HIVȒ	TSPO PET correlates with NP assessment in specific domains	

BBB, blood–brain barrier; cART, combined antiretroviral treatment; CSF, cerebrospinal fluid; CT, computed tomography; CVD, cardiovascular disease; DNA, deoxyribonucleic acid; DTI, diffusion tensor imaging; FA, fractional anisotropy; FC, functional connectivity; FDG, fluorodeoxyglucose; fMRI, functional MRI; Glx, glutamine/glutamate; GM, grey matter; HAD, HIV-associated dementia; HAND, HIV-associated neurocognitive disorder; HIV, human immunodeficiency virus; IP-10, interferon gamma-induced protein 10; MCP-1, monocyte chemoattractant protein-1; MD, mean diffusivity; MI, myo-inositol; MIP-1α, macrophage inflammatory protein-1α; MIP-1β, macrophage inflammatory protein-1β; MND, mild neurocognitive disorder; MRI, magnetic resonance imaging; MRS, magnetic resonance spectroscopy; NAA, N-acetylaspartate; NP, neuropsychological; PBMC, peripheral blood mononuclear cell; PET, positron emission tomography; RNA, ribonucleic acid; sCD14, soluble CD14; TSPO, translocator protein; VEGF, vascular endothelial growth factor; WM, white matter.
